# A technical note on variable inter-frame interval as a cause of non-physiological experimental artefacts in ultrasound

**DOI:** 10.1098/rsos.170245

**Published:** 2017-05-31

**Authors:** D. Miguez, E. F. Hodson-Tole, I. Loram, P. J. Harding

**Affiliations:** School of Healthcare Science, Manchester Metropolitan University, Manchester, UK

**Keywords:** synchronization, temporal behaviour, tracking, tendon, frame timing correction, medical imaging

## Abstract

Ultrasound (US) imaging is a well-recognized technique for the study of static tissues but its suitability for studying tissue dynamics depends upon accurate frame time information, which may not always be available to users. Here we present methods to quantify the inter-frame interval (IFI) variability, and evaluate different procedures for collecting temporal information from two US-imaging devices. The devices tested exhibited variable IFIs that could only be confirmed by direct measures of timing signals, available by means of electrical signals (triggers) and/or temporal information contained in the software used for the US data collection. Interpolating frame-by-frame measures of dynamic changes within image sequences using individual IFI values provided improved synchronization between measures of skeletal muscle movement and activation; validating US as a valuable technique for the study of musculoskeletal tissue dynamics, when correctly implemented.

## 1. Introduction

Since the first two-dimensional ultrasound (US) scans (b-mode) were obtained [1,2], US imaging has evolved to become a valuable tool for studies of features of static biological tissues (e.g. muscle, tendon and tumours). There is increasing use of US image sequences to measure features of tissue displacement. For example, in the musculoskeletal system, changes in fascicle orientation, strain and strain rate [3–5] are commonly reported and recent reports suggest measures of muscle tissue displacement within localized regions of the image may be valuable for diagnosis and monitoring of neurodegenerative diseases [6]. In studies aiming to quantify patterns of movement in the imaged tissue (e.g. in relation to voluntary activation), or link movement patterns to externally measured parameters (e.g. joint torque), the precise time at which each frame in an image sequence is collected is imperative.

Conventional US devices typically display and record the frame rate as a single value, based upon the specific configuration of the machine for a certain experiment. This masks variation in the inter-frame interval (IFI) or frame rate (frame rate = 1/IFI) during the acquisition. In recent years, some manufacturers have begun providing access to additional temporal information, forming part of the headers of the collected frames (usually stored in proprietary file formats). To our knowledge, the potential value of this additional information has not been explicitly reported with respect to dynamic imaging.

Problems owing to synchronization between US and other data acquisition devices (e.g. dynamometers and electromyography) have previously been reported [7]. The focus of these discussions has however been on the potential offset between systems at initialization of data acquisition. For example, Finni *et al*. [2] demonstrated how increasing the offset between US-derived tendon length and dynamometer measured force significantly altered measures of elastic hysteresis in human Achilles tendon, with implications for understanding the ability of human tendon to dissipate energy owing to its material viscosity. Synchronization may however also result from variable IFIs, a factor which, to our knowledge, has not previously been specifically investigated.

The work presented here therefore addresses two key questions:

— to what extent is IFI constant within a sequence of US images? and
— to what extent can accounting for variable IFIs provide better synchronisation between experimental data measures?

Two methods of measuring IFI in US devices are presented to address these questions and demonstrate the need to establish appropriate synchronization across devices for different experimental conditions.

## 2. Material and methods

### 2.1. Demonstration of effects of variable inter-frame intervals

To demonstrate the effects of assuming a constant IFI, both US and surface myoelectric (EMG) data were recorded from a skeletal muscle while small involuntary muscle twitches were evoked using low-level electrical stimulation of the motor nerve. A constant IFI would provide a consistent, constant relationship between electrical stimulation and the resulting muscle activation and tissue displacement seen in the collected US sequence.

#### 2.1.1. Data collection

US (LogicScan 128, Telemed Ultrasound Medical Systems, Italy) and EMG (Trigno Wireless EMG, Delsys Inc, USA) data were collected from the medial gastrocnemius muscle of the left leg of a single participant. The participant provided written informed consent and the study was approved by the Faculty of Science and Engineering Ethics committee at Manchester Metropolitan University.

The US probe (linear array, 59 mm long, 7 MHz latent frequency) was aligned with the fascicle plane and held in position using elasticated bandage. The EMG electrode was placed immediately alongside the US probe, on a patch of skin that had been shaved and cleaned of surface debris. For electrical stimulation, an anode (DEL001, Biosense Medical LTD, USA) was placed over the patella and cathode (Bluesensor SP, Ambu, Denmark) placed over the tibial nerve in the region of the popliteal fossa.

**Figure 1.**
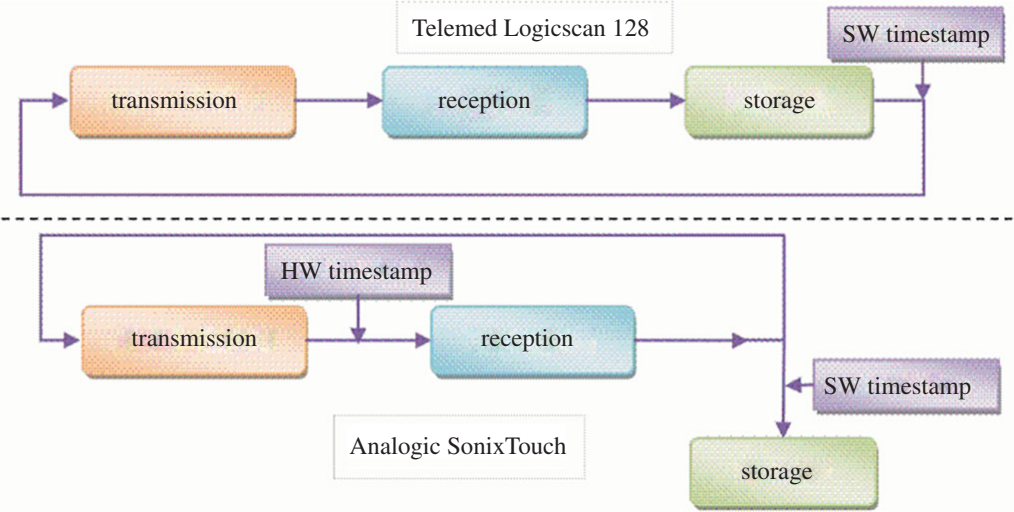
Block diagram of the process of ultrasound (US) transmission, reception and data storage proposed to occur in the two US machines analysed, Telemed Logicscan 128 (top) and Analogic SonixTouch (bottom). Note the linear arrangement in the Telemed process, which underpins accumulation of time delays when a constant IFI is assumed. The approximate positions of software (SW) and hardware (HW) timestamp signals collected and studied are indicated at appropriate points in the cycle.

US at both 42 fps and 82 fps (the frame rate stated on the US machine during collection), and EMG data were collected for 40 s, during low-level electrical stimulation (DS7, Digitimer, UK, 12 mA, 200 µs pulse width, 1 pulse every 5 s) of the nerve (figure 1). US imaging, EMG and stimulation were synchronized using a common trigger, controlled via the Matlab data acquisition toolbox (R2014a, Mathworks, USA). EMG was collected at a rate of 2 kHz through an analogue acquisition card (USB-6211, National Instruments, USA).

#### 2.1.2. Data analysis

To identify the IFIs, metadata were extracted from the proprietary files (Telemed Video Data files, extension:.tvd) using a utility made freely available by the US device manufacturer (b to lines, Telemed, Italy). Extracted data provide many specifics about the parameters of transmission, reception and, most importantly, the IFIs accurate to 100 ns (figure 1).

To identify the occurrence of the electrically evoked muscle twitches in the recorded US images a KLT feature tracking [8] and mutual information based approach was applied, exactly as described by Harding *et al*. [4]. Briefly, the KLT tracker estimates movement between consecutive images. It first classifies image regions by calculating a measure of their likelihood to be accurately identified in the subsequent frame of the sequence [9]. The most ‘trackable’ image regions (known as *features*) are then tracked into the next frame of the image sequence. Each feature undergoes an iterative search procedure which attempts to minimize the differences between the feature selected in frame one and the new position of this feature in frame two [8]. The result of this process is a set of vectors describing the magnitude and the direction of movement at multiple points across the image.

To identify the presence of a ‘muscle twitch’ between two frames, the movement vectors were analysed using a mutual information analysis [10]. This has been shown to differentiate between small, random, noise like movements of features within US images, and more coherent, structured movements; such as those of muscle twitches resulting from electrical stimulation [11]. The result of this analysis is a frame-by-frame measure representing the likelihood that a muscle twitch is present at any given frame.

**Figure 2.**
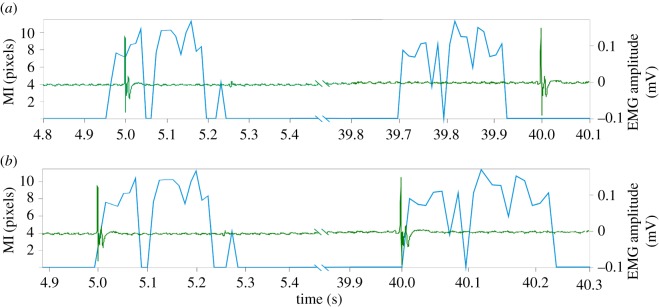
Illustration of data collected during low-level electrical stimulation of *medial gastrocnemius* in a healthy, adult volunteer: surface EMG (green) is plotted together with the results of mutual information (MI) applied to the KLT features extracted from the ultrasound images (blue); the first and the last stimuli (at 5 and 40 s, respectively) are shown when: (*a*) assuming a constant frame rate of 82 fps and (*b*) using the time information contained in the frame headers provided by the manufacturer's proprietary software.

The EMG data were plotted against mutual information results to demonstrate the effects of assuming a constant IFI (figure 2*a*). This figure is plotted on the assumption that EMG data were sampled at a constant rate of 2 kHz (set via the data acquisition toolbox) and US was sampled at a constant rate of 82 fps (frames per second = 1/IFI), as stated by the US device display.

### 2.2. Comparison of hardware and software based time stamp information

In a separate experiment, the behaviour of two different methods for logging US system frame timing (timestamp) was investigated. The methods compared were the collection of hardware generated timing signals and software timestamp data (HW and SW, respectively, in figure 1, bottom panel). The machine used during this experiment was a SonixTouch US device (Analogic, *previously Ultrasonix*, BK Ultrasound, USA). Data were collected for 10 s per trial. No US images were analysed during this experiment, as the focus was solely to investigate the difference in timestamping methods. In order to evaluate the timestamping performance at different frame rates, the number of elements within the transducer used for receiving the rebounding US waves was manually modified varying from 64 (approx. 150 fps) to 256 (approx. 37 fps).

#### 2.2.1. Hardware timing signal acquisition

**Figure 3.**
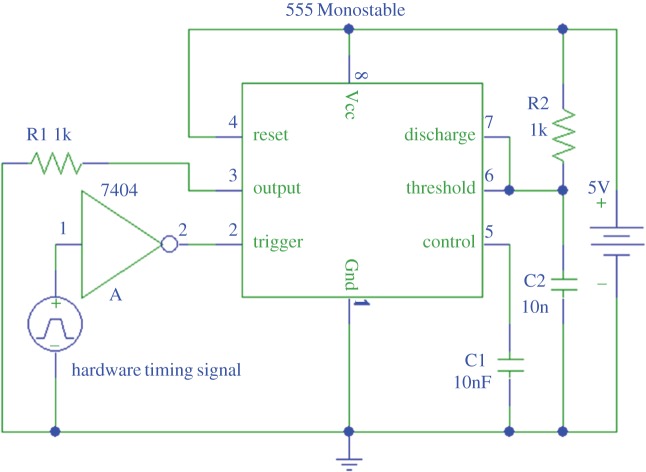
Schematic of the electronic circuit designed and implemented for extending the pulse width of the hardware timing signal to a rate achievable by the used data acquisition card (USB-6212, National Instruments, USA), consisting of a 555 Monostable timer activated by the inverse (7404 NOT gate) of the hardware timing signal.

The hardware timing signal is a pulse signalling the end of the ultrasonic transmission of every frame; it consists of a differential TTL signal with a 25 ηs pulse length, equivalent to one cycle of the US hardware's internal clock (40 MHz). A signal of such a short length (25 ηs) could not be reliably acquired by our data acquisition card (USB-6212, National Instruments, USA), which operates at a maximum of 400 kS s^−1^. To overcome this, an electronic circuit was designed and implemented for extending the pulse width of the US device hardware timing signal (figure 3). The circuit consists of a 555 timer, in a monostable configuration, with an inversion of the input signal (the hardware timing signal) using a NOT logic gate. This circuit both amplified and extended the duration (24 µs pulse width) of the hardware trigger making it measurable by our acquisition card.

#### 2.2.2. Software time stamping

The US machine was controlled by customized software developed in C++ based on the Ulterius software development kit (Analogic, *previously Ultrasonix*, BK Ultrasound, USA). Ulterius provides a method by which the number of frames transmitted since the start of acquisition can be obtained, and the time each US frame was made available to software was recorded from the processor clock (precision: 3.579 GHz → 27.937 µs) of the PC.

During data acquisition, the IFI from both the hardware timing signal and the software timestamps were recorded simultaneously. In addition, a control experiment was carried out, in which IFIs were recorded at three frame rates but no data was copied to local memory. This provided a condition during which memory consumption and processor overhead were reduced to their absolute minimum, ensuring that the transfer and storage of data could not cause the variation in the IFIs recorded.

## 3. Results

### 3.1. Inter-frame interval variations

Evidence of IFI variability is demonstrated with the simultaneous acquisition of US and EMG data recorded from medial gastrocnemius muscle during low-level electrical stimulation of the tibial nerve. Figure 2*a* illustrates the effects of assuming a constant IFI, and shows that the time difference between the application of a stimulus and the resulting muscle activation is 49 ms, greater than the IFI (12.2 ms), even from the very first stimulation at *t* = 5 s. The difference at *t* = 40 s increases to over 300 ms, equivalent to more than 24 times the IFI. This error is so great that the muscle movement appears to *precede* the electrical stimulus, an electrophysiological impossibility.

The IFI values extracted from metadata confirmed the experimental findings, and were actually found to follow a bi-modal distribution, regardless of the imaging parameters used. At 82 fps, the majority (92.72%) of the frames clustered around a frame rate of 83.5 fps, while the remaining frames clustered around 62.5 fps. When data were recorded at 42 fps the majority (85.62%) of the frames were clustered around approximately 41.5 fps whereas the rest clustered at 36 fps. The resulting mean frame rates were 81.83 fps ± 5.38 (mean ± standard deviation, *n* = 17 761 frames) and 40.81 ± 2.086 fps (*n* = 9984 frames), respectively.

The application of frame specific IFI values can be seen in figure 2*b*, which shows how the timing information extracted from the headers of the US image files corrects the synchronization errors seen in figure 2*a*. This demonstrates that the timing correction performed did reduce the measureable delay between the electrical stimulation and mechanical activation, so that it is consistently shorter than the IFI (82 fps, or an IFI of 12.2 ms), the greatest possible accuracy for this experiment.

### 3.2. Comparison between hardware and software based time stamp information

**Figure 4.**
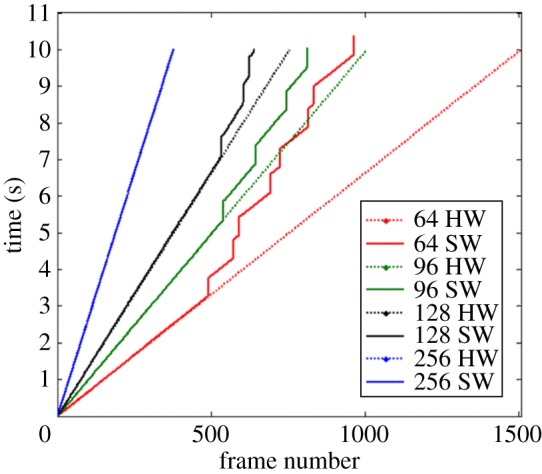
Hardware (dotted lines) versus software (solid lines) frame time stamps from 10 s long acquisitions using 64, 96, 128 and 256 elements (resulting in 150.7, 100.5, 75.4 and 37.7 fps frame rates respectively) for the Analogic SonixTouch machine.

**Figure 5.**
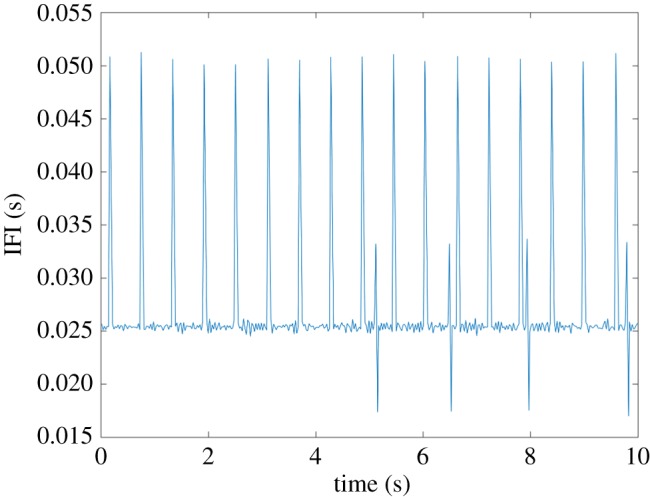
IFIs calculated from software timestamps from a 10 s duration acquisition using 256 elements (resulting in 37.7 fps frame rate) for the Analogic SonixTouch machine. A bimodal distribution of IFIs comprising approximately 0.025 and approximately 0.05 s is evident in the first 5 s of acquisition, with more variability occurring between 5 and 10 s.

Comparison of the IFI values obtained from capturing the hardware timing signal and the software timestamps also showed inconsistencies (figure 4). Clear discontinuities in the IFIs from software timestamps were evident in all but the slowest frame rates tested, meaning that frames were ‘dropped’, i.e. not able to be collected. When data were collected at the slowest frame rate (37 fps) although no frames were dropped (figure 4), closer inspection of the software timestamp signal reveals inconsistency in the IFI did still occur (figure 5). By contrast, IFIs from hardware timing signals are shown to be constant across the length of each trial (figure 4), which confirms that US transmission did take place, regardless of whether image data were made available for collection or not.

During tests when no data were transferred to the local memory, i.e. when processing time and memory consumption were reduced to the bare minimum, no frames were dropped for any of the analysed frame rates. The software timestamps and hardware timings signals collected during these experiments were still found to differ though. The distribution of the hardware timing signal exhibited a variance lower than the measurement accuracy at which the data were collected (25 µs) meaning the transmission was constant in time. The software timestamps however show a great deal of variance, proportionally increasing with frame rate. This shows that, even with minimal computational overhead, the IFIs recorded via software timestamps are not as consistent as those recorded by the hardware timing signal.

## 4. Discussion

This work addresses two key questions posed in relation to variations in IFI in recorded US image sequences. We have shown from empirical data (figure 2), for the first machine tested (Telemed Logicscan 128), that assuming a constant IFI can lead to dissociation of measurements; to such an extent that a muscle is shown to respond to an electrical stimulation prior to the delivery of the stimulus (figure 2*a*). While this demonstration provides a somewhat extreme example, it illustrates the consequences of the inappropriate relationships (mechanical response of muscle preceding electrical activation) which can be observed if variations in IFI are not accounted for. One approach to mitigate these issues is the reduction of the acquisition duration; however, we observed that within a US sequence of just 5 s the error had accumulated to more than four times the IFI (49 ms). A second approach, which we implemented, is to employ the accurate timing data provided by the US device (figure 2*b*). This option however, depends on the provision of that utility by the manufacturer and therefore may not be available across all commercial devices. The facility to access accurate frame time information should therefore be a consideration when new devices are purchased for the laboratory, particularly if tissue dynamics are likely to be the focus of any studies. For cases when equipment is already present in the laboratory, but accurate frame timing information is not available from the device, we have provided a Matlab utility for performing the analysis described in the methods, available from http://uk.mathworks.com/matlabcentral/fileexchange/59006-ultrasound-find-best-framerate or by contacting the authors directly. The code enables users to assess whether or not data, collected using the stimulated muscle twitch protocol described, were acquired by a US machine with a non-constant IFI and allows the correction of constant delays present in the data collection.

In the case of the second machine tested (Analogic SonixTouch), the observed frame losses in the software timestamps show that, while US beam transmission was consistent (figure 4), it was not possible to save all frames to memory. The frame loss is inversely proportional to the IFI, indicating that the system memory saturated at rates proportional to the amount of frames collected. Despite these issues, variations in the IFIs (frame loses) recorded by software timestamping were found to have zero mean (i.e. they were non-cumulative), therefore to assume a constant IFI was valid. This said, the fact that frames could be lost during an acquisition must be accounted for to ensure synchronization is maintained throughout the duration of an experiment. In the work presented here, the identification of the frame losses was achievable via the control software, but this could also easily be achieved via the comparison of software timestamps and the hardware timing signal. The frame losses have wider implications when extracting dynamic information from US data. During tissue displacement measurements, these ‘jumps’, if not accounted for, would result in a twofold increase in the resulting measurements (assuming constant movement).

In summary, we have shown that the stated temporal consistency of US devices cannot necessarily be relied upon. In tests of two machines, we have demonstrated that one machine (Telemed Logicscan 128) has highly variable IFIs, but provides a method by which reliable IFIs may be extracted from recorded US sequences. By contrast, the second machine (Analogic SonixTouch) was shown to have an IFI that was indeed constant (or at the least had a lower variability than our measurement accuracy) but where the most accessible method by which IFIs could be recorded (SW timestamp was found to reveal *false variability* (figure 4). In the case of this machine, while transmission and reception were constant, hardware limitations resulted in some frames being irretrievable owing to memory saturation (figure 1).

Consideration of these findings in the design and implementation of experiments is critical for the reporting of reproducible data and interpretation of results in relation to physiological phenomena. Our results show that synchronously starting data acquisition across multiple devices (e.g. US, electromyography, dynamometry) is not adequate for correct intra-trial synchronization, a factor which may have contributed to the discrepancies in tendon hysteresis values reported between animal and human studies [12]. Reduced intra-trial synchronization may also be a contributing factor in the variation in muscle behaviour identified using other US modalities (e.g. large variations reported in the delay between EMG determined activation and tissue doppler/motion mode based muscle movement reported across two recording days as: −20.3 ± 21.0 ms and 17.4 ± 27.2 ms, [13]). The importance of intra-trial synchronization is also critical when considering recent development of computational US image analysis techniques for applications such as diagnosis and monitoring of features of neurodegenerative diseases [6]. In that report, computational analysis provided an automated means of detecting tissue displacements (twitches) resulting from involuntary activations (fasciculations), but the work should be extended to evaluate performance of the approach against recorded myoelectric signals (current clinical gold standard), which will need to be recorded in a fully synchronous manner. In addition, the work could be extended to provide additional biomarkers of disease progression by quantifying the spatial and temporal features of recorded twitches where, again, intra-trial synchronization will be an important factor to consider.

## 5. Conclusion

We conclude that the study of tissue dynamics using US technology should include an investigation of the frame rate variability within the experimental set-up, prior to the data collection. This work demonstrates methods by which researchers can gain a more complete understanding of the temporal behaviour of specific US devices. This will serve to both improve the accuracy of dynamic measurements using US technology, and support the reporting of reproducible data.

## Data accessibility

Data from this study are available from the Dryad Digital Repository: (http://dx.doi.org/10.5061/dryad.550sg) [14].
